# Microfabricated Formaldehyde Gas Sensors

**DOI:** 10.3390/s91109196

**Published:** 2009-11-18

**Authors:** Jonas Flueckiger, Frank K. Ko, Karen C. Cheung

**Affiliations:** 1 Department of Electrical and Computer Engineering, University of British Columbia, Vancouver BC, V6T 1Z4, Canada; E-Mail: kcheung@ece.ubc.ca; 2 Department of Materials Engineering, University of British Columbia, Vancouver BC, V6T 1Z4, Canada; E-Mail: frank.ko@ubc.ca

**Keywords:** formaldehyde sensor, MEMS, conducting polymer sensor

## Abstract

Formaldehyde is a volatile organic compound that is widely used in textiles, paper, wood composites, and household materials. Formaldehyde will continuously outgas from manufactured wood products such as furniture, with adverse health effects resulting from prolonged low-level exposure. New, microfabricated sensors for formaldehyde have been developed to meet the need for portable, low-power gas detection. This paper reviews recent work including silicon microhotplates for metal oxide-based detection, enzyme-based electrochemical sensors, and nanowire-based sensors. This paper also investigates the promise of polymer-based sensors for low-temperature, low-power operation.

## 1. Introduction

Effective detection of chemicals in the environment requires a simple, rapid, sensitive and selective analytical sensor. Such devices could continuously monitor our surroundings and give us warnings about the level of toxic chemicals in our workplaces, factories, and homes, even when they are present in extremely low concentrations.

Formaldehyde is often a component in urea-formaldehyde adhesive resins which bind pressed wood products such as plywood, veneers, and particle board. Formaldehyde is also used in the manufacture of paper, textiles, and paints. However, formaldehyde is a hazardous air pollutant and prolonged exposure to formaldehyde can cause serious health effects. Formaldehyde has been linked to cancer deaths; recent findings show that factory workers who had been exposed to high formaldehyde levels were at increased risk for leukemia [1]. In the home, off-gassing of formaldehyde over time from pressed wood products can also pose health hazards. Indoor, non-industrial exposure to chemical hazards can occur continuously at low levels, contributing to symptoms such as headaches, fatigue, and upper respiratory and eye irritation. In Japan, energy-saving homes and buildings have increased airtightness to reduce energy costs, and the reduced ventilation can lead to prolonged exposure to outgassing chemicals from the plywood, particle board, and insulating materials, and an increase in occurrence of “sick house syndrome” [2]. The occurrence of “sick-building syndrome” [3], also comprising these non-specific but acute health effects, has been linked to indoor chemical contaminants such as formaldehyde from adhesives, upholstery, and manufactured wood products [4-6]. Formaldehyde is considered a major contributor to sick building syndrome. Formaldehyde is also formed during ozonation as part of some water pre-treatment processes, and as a natural metabolite which can accumulate in some species of frozen fish [7]. Formaldehyde levels of 1–3 ppm can cause irritation in the eyes and nose, and levels above 10 ppm cause strong discomfort. In North America, current safety standards limit the maximum exposure to 2 ppm over an 8-hour average, while indoor levels should not exceed 0.08 ppm (80 ppb) over 30 minutes in the home. The Chinese Environmental Protection Agency limits exposure to 0.06 ppm over 30 minutes [8].

Continuous monitoring of formaldehyde levels in the environment would require stable sensors with long lifetime. A low detection limit (sub-ppm) is required for monitoring long-term safety, and high selectivity is necessary in the presence of other interferents. The deployment of distributed sensor arrays in factories and homes would require low-power devices. In this review, we focus on microfabricated sensors which promise to address some of these issues, including portability and power consumption. We also examine advances in nanotechnology and polymer technology which promise to bring further improvements in lower detection limits, decreased power consumption, and increased selectivity.

### 1.1. Current Methods for Formaldehyde Detection

Several currently available techniques to measure gaseous formaldehyde require the vapor to be adsorbed onto a filter or into a liquid solution, which is then further analyzed using methods such as electrochemical detection, ion chromatography, high performance liquid chromatography [9], voltammetry [10,11], or photometric or fluorometric determination. These methods are not suited for real-time monitoring, and they often require large, expensive laboratory equipment.

Bioelectronic sniffers for gaseous formaldehyde have been developed which incorporate immobilized enzymes such as aldehyde dehydrogenase (ALDH) or formaldehyde dehydrogenase (FALDH) [12,13]. The enzyme electrodes are located in a liquid compartment which is separated from the gas compartment by a diaphragm. In this way, formaldehyde in the gas phase can be detected amperometrically [14]. Formaldehyde dehydrogenase requires the presence of a co-factor, β-nicotinamide adenine dinucleotide, which enables formaldehyde conversion. Storage stability of enzyme-based sensors may be an issue. Kataky *et al.* reported a disposable FALDH-based sensor with screen-printed electrodes [15]. The enzyme and co-factor were placed behind a polyurethane membrane, and this device had a 50% loss in sensitivity after two weeks when stored at 4 °C, but <10% decrease in response after two weeks when stored at room temperature. Achmann *et al.* used a Teflon membrane to separate the liquid phase from the gas phase and phenothiazine (PT) as a mediator to detect the formed NADH [13,16]. They reported a linear response in the tested range (1–15 ppm) with a sensitivity of 1.9 μA/ppm and a detection limit of about 130 ppb. The sensor showed no significant response to other tested chemical gases. The group later modified the sensor and increased the sensitivity to 76 ppb [17]. Korpan *et al.* used the enzyme alcohol oxidase (AOX) to develop a potentiometric formaldehyde sensor using a field-effect transistor which had stable response for more than 60 days when stored at 4 °C [7].

Gaseous formaldehyde can also be directly measured using cataluminescence [18], chemiluminescence [19], colorimetry [20,21], infrared absorption [22], amperometric detection [23-25], or using sensors incorporating semiconductor metal oxide thin films [26], metal oxide films [27,28], or metal oxide nanoparticles [29]. Portable units have detection ranges from 0–10 ppm, but at low concentrations (<0.1 ppm) there is significant cross-sensitivity to alcohols and other interferents. Although some metal oxide sensors operate at temperatures as low as 95 °C [26], the more typical operating range is from 300 to 400°C, thus requiring high power consumption [30].

To date, the majority of microscale formaldehyde sensors have relied on metal oxides as the sensing material. In metal oxide sensors, electron donors or acceptors in the gas phase adsorb onto the metal oxide. At high temperatures (>200 °C), the adsorbed species can exchange electrons with the metal oxide: an acceptor molecule will take electrons and reduce its conductivity, while an electron donor will increase the conductivity. These surface interactions are thermally activated. The absorption probability of the gas onto the surface can be predicted by a Langmuir isobar which limits the sensor operation at the high-temperature side, and the surface combustion of the adsorbed analyte limits the sensor operation at the low temperature side. The spontaneous desorption of the gas from the surface also has a minimum thermal energy [30]. These effects combine to determine the temperature window of operation for a given material. Commonly used materials include SnO_2_, TiO_2_, and WO_3_, which are metal oxide semiconductors. These materials are typically thick films in commercially available sensors. Many authors report as well an enhanced response magnitude when doping the metal oxides with metal nanoparticles [31-33]. The underlying interaction between the gas and the solid involves the catalytic oxidation of the target gas over the surface. In most cases adsorbed oxygen is utilized for the oxidation reaction and the consumption of surface oxygen reduces the surface space charge which in turn is manifested in a change in electrical conductivity. If the bare oxide is not active enough the presence of metal particles such as Au, Pd, Pt, Al enhances the adsorption/desorption properties of oxygen on the metal oxide surface and therefore as well the catalytic oxidation of gases.

## 2. Microscale Formaldehyde Sensors

### 2.1. Metal Oxide Based Sensors

**Figure 1.**
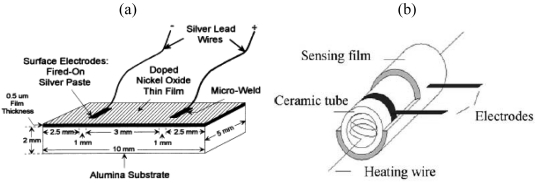
Formaldehyde sensor configurations: (a) ceramic plate structure, reprinted from [27] with the permission from Elsevier and (b) ceramic tube structure, reprinted from [8] with permission from Elsevier.

Sensors based on metal oxide semiconductors are inexpensive and relatively easy to use: they convert a chemical concentration directly into an electrical output by simply measuring the resistivity of an exposed thin film [30]. A key issue when using metal oxides is the requirement of elevated temperature of operation. The most simple approach is using an external heat source [27,34]. The substrate under the thin sensing film is placed on a heated substrate, or hotplate (Figure 1a). Although this setup is not suitable for direct miniaturization, it remains very useful for characterizing thin films.

Other work was done by depositing the sensing film onto a ceramic tube [35,36] with electrodes and a heating wire (Figure 1b). This ceramic tube structure can be welded onto a standard electronic packaging base element. The temperature is controlled by the current flowing through the heating wire. Xu *et al.* used this configuration to detect formaldehyde gas with a mixed oxide of ZnO/ZnSnO_3_ doped with Au [32]. They found that exposure to 50 ppm formaldehyde induced a 34.5 times change in the conductivity; the sensor also had high selectivity against ammonia, benzene, toluene, and other interfering gases. Huang *et al.* [35] used a sol-gel process to dope rare earth oxides LaFeO_3_ with Zn. The obtained powder was dispersed in a polyvinyle acetate (PVA) solution and then coated onto the ceramic tube (Al_2_O_3_). To improve stability and repeatability the tube was calcinated at 400 °C for 2 h. Zhang *et al.* [36] used the same ceramic tube and LaFeO_3_ powder but doped it with Pb [36]. Chen *et al.* fabricated CdO-In_2_O_3_ powder through calcinations on ceramic tubes at different temperatures [26,37]. They report the best sensing properties when calcinated at 650 °C of about an 80 fold increase in electric resistivity when exposed to 10 ppm of formaldehyde. They later doped the CdO-In_2_O_3_ film with SnO_2_ to further increase the sensitivity [37]. The improved sensing layer showed a sensitivity defined as the ratio of the electrical resistance in air to that in gas of 559 when exposed to 300 ppm formaldehyde.

Wang *et al.* used the ceramic tube structure to characterize the sensing properties of SnO_2_ doped with hydroxyl-functionalized multi-wall carbon nanotubes (MWCNTs) [8]. They report a much higher response to formaldehyde than that of undoped SnO_2_. The lowest concentration of formaldehyde detected by a 5 wt% MWCNT doped SnO_2_ sensing layer was 0.03 ppm. They explain the increase in sensitivity with the high adsoption capacity of MWCNTs. The material showed a response when exposed to acetone, methanol, toluene, benzene and ammonia. However the response was twofold stronger towards formaldehyde.

#### 2.1.1. Microfabricated integrated hot plates

Silicon based microfabrication offers the possibility to miniaturize and integrate the heating element using the same processes as those involved in fabricating the control circuitry. The sensing element must be independently heated and thermally isolated from the other parts of the device. The power consumption as a function of operating temperature for microfabricated silicon hotplates is in the range of 50–150 mW at 400 °C, depending on the geometry [38]. The silicon hotplates, which have low thermal mass, can be fabricated using SOI wafers or bulk micromachining (KOH etching or reactive ion etching) [39]. This power consumption is much lower than the typical 0.5–1 W required for conventional metal oxide sensors operating at 400°C in which the sensing materials are printed onto ceramic substrates [40]. The small thermal mass also permits sensor operation in a dynamic mode on the millisecond time scale, using short temperature pulses and measuring response signatures which are specific to different gases [41].

**Figure 2.**
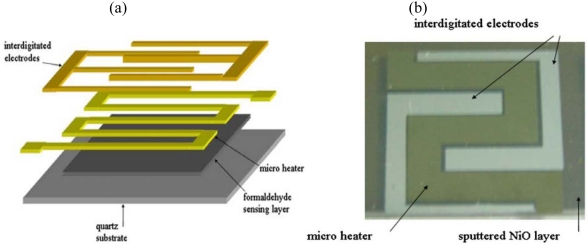
Formaldehyde sensor with integrated Pt micro-heater and Au interdigitated electrodes on a sputtered NiO layer, reprinted from [42] with the permission of Springer Science + Business Media.

Lee *et al.* used microfabrication techniques to pattern a platinum resistive heater on top of a 2 μm-thick sputtered NiO sensing layer on a quartz substrate for formaldehyde detection [42] (Figure 2). Gold interdigitated electrodes were then patterned using Cr as an adhesion layer. The gold electrodes were used to measure the conductivity change in the NiO film upon adsorption of formaldehyde. The micro-hotplate was operated from 150–280 °C, with the lowest detection limit at 1.2 ppm at 280 °C and average time constant of 13 seconds in the 4.0–8.0 ppm concentration range. To further decrease the power consumption they improved the thermal insulation of the sensing element by using bulk micromachining to fabricate a suspended silicon nitride membrane. NiO was then sputtered on top of this membrane. They achieved better sensitivities compared to their previous work by controlling the substrate temperature during the sputtering process leading to a preferred orientation of the oxide crystals [43]. This sensor had a lowest detection limit of 0.8 ppm at 300 °C for a 0.4 μm-thick NiO film.

Wang *et al.* used the same configuration and found that co-sputtering NiO/Al_2_O_3_ could improve the sensitivity [44]compared to NiO. They also decreased the thickness of the sensing layer, and developed a second configuration in which the NiO/Al_2_O_3_ was sputtered on top of the electrodes, thus increasing the area of the sensing layer that is in contact with the environment.

**Figure 3.**
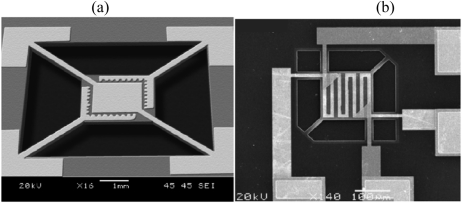
Formaldehyde sensor with integrated Pt micro-heater on a suspended silicon nitride structure, reprinted from [43] with the permission from Elsevier and on a suspended SiO_2_/SiN_x_/SiO_2_ membrane, reprinted from [28] with permission from Elsevier.

**Figure 4.**
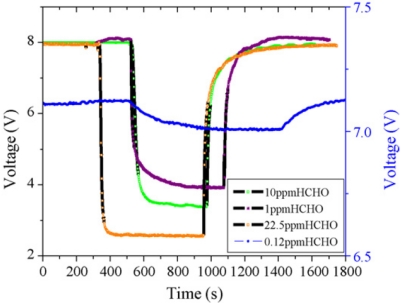
Response curves as a function of formaldehyde concentration in a micro-hotplate metal oxide sensor, reprinted from [28] with permission from Elsevier.

Lv *et al.* used surface micromachining to define the microhotplate and a SnO_2_–NiO composite film as the sensing layer [28] (Figure 3b). The lowest detection limit was 0.06 ppm, with 180 mW power consumption at 300 °C. They found that the response time decreases rapidly with increasing formaldehyde concentration (from ∼200 s at 0.12 ppm to ∼20 s at 10 ppm) but then plateaus above 10 ppm because the sensor is limited by the reaction rate of formaldehyde and adsorbed oxygen at the sensor surface (Figure 4).

The use of self-heating metal oxide nanowires or nanocrystals can also reduce power consumption. Using individual SnO_2_ nanowires which were pre-synthesized and then transferred onto platinum electrodes, Prades *et al.* demonstrated that the response to NO_2_ was similar when operated in self-heating mode and when operated with an external microheater [45]. The wire self-heats through the Joule effect and the power dissipated is given by *P = I^2^R* where *I* is the current and *R* is the nanowire resistance. Their maximum power dissipated was ∼27 μW for 300 nA current, which gave a temperature of ∼300 °C. Sensors based on this principle will require calibration to account for the change in nanowire resistance with increasing temperature and with exposure to different gas concentrations.

### 2.2. Metal Oxides for Photocatalytic Detection of Formaldehyde

The wide-scale deployment of metal oxide sensors operating at high temperature in large area sensor networks may in future be limited due to their high power consumption. Some gas sensors have also been found to have gas sensitivity at room temperature under ultraviolet (UV) illumination [30], and the mechanism for photoinduced reactivity of semiconductors such as TiO_2_ has been widely studied [46,47].

Metal oxides such as TiO_2_, ZnO, and WO_3_ exhibit a small and slow low-temperature gas sensitivity that is enhanced with UV light. Under UV illumination, the photons have sufficient energy to excite valence band electrons to the conduction band, which then migrate to the surface of the solid. These electrons participate in reactions with molecules adsorbed at the solid surface. TiO_2_ is one of the most commonly used photocatalysts, with anatase (E_g_ = 3.23 eV) and rutile (E_g_ = 3.02 eV) crystal forms commonly used for this application. It can be activated by near UV illumination (300–370 nm) as well as UV light (254 nm), and it can degrade a broad range of molecules for use in indoor air purification [48], including alkanes, ketones, alcohols, phenols, and aromatic compounds [49]. The photodegradation of formaldehyde results in carbon dioxide [50]:

$$\begin{array}{c} {\text{TiO}}_{2}\overset{h\nu }{⟶}e^{-}+h^{+}\\ {\text{H}}_{2}\text{O}\to {\text{H}}^{+}+{\text{OH}}^{-}\\ h^{+}+{\text{OH}}^{-}\to {\text{OH}}^{•}\\ e^{-}+{\text{O}}_{2}\to {\text{O}}_{2}^{-}\\ \text{HCHO}+{\text{OH}}^{•}\to {\text{HCO}}^{•}+{\text{H}}_{2}\text{O}\\ {\text{HCO}}^{•}+{\text{OH}}^{•}\to \text{HCOOH}\\ \text{HCOOH}+2h^{+}\to {\text{CO}}_{2}+2{\text{H}}^{+} \end{array}$$

This reaction requires the presence of oxygen as well as water adsorbed at the metal oxide surface. The oxygen captures TiO_2_ electrons and assists in charge separation [51], preventing electron-hole recombination. The adsorbed water is oxidized to produce hydroxyl radicals OH͘ which are the primary oxidant [52]. The adsorbed water also enhances oxygen photoadsorption by trapping photogenerated holes at OH^-^ sites [53]. The production of stable reaction intermediates can stop the reaction by adsorbing to the catalyst and blocking reaction sites on the TiO_2_. In the case of formaldehyde, the formic acid intermediate is converted to CO_2_ after further UV illumination.

Obee and Brown [54] found that increasing the humidity level increased the TiO_2_ oxidation rate of formaldehyde up to a maximum, then decreased with increasing humidity for concentrations in the range of 10 ppm and above. For low formaldehyde concentration, 1.5 ppm and below, the oxidation rate had a weak dependence on humidity. The oxidation rate also depended on the formaldehyde concentration, increasing to a maximum at ∼100 ppm. The formaldehyde photo-oxidation rate decreased with increasing temperature.

The presence of other interferents in the air will lead to competitive interaction at the TiO_2_ surface adsorption site. Noguchi *et al.* [55] found that oxidation rate of formaldehyde was higher than that of acetaldehyde because TiO_2_ has higher adsorption capacity for formaldehyde; Obee and Brown [54] found that formaldehyde is more strongly adsorbed than toluene. Ao *et al.* [50] worked with VOC concentrations in the 20–200 ppb range, which better reflect levels commonly found in the indoor environment. They found that the photodegradation rate of formaldehyde was reduced in the presence of other VOCs such as benzene, toluene, ethylbenzene and *o*-xylene, because the photodegradation intermediates of these molecules blocked the active surface sites on the TiO_2_.

Thick film TiO_2_ sensors were developed which used the photocatalytic response to different organic molecules in the gaseous phase [56]. The cermet sensors operated at room temperature and the adsorption of different gases to the surface gave different resistivity signatures. The sensors were regenerated by exposing to ambient air for 24 hours and did not require heat.

Peng *et al.* have used ZnO nanorods to detect formaldehyde under UV illumination [57]. The nanorods were deposited onto ITO electrodes on a glass substrate. They measured increased conductivity in the nanorod due to photocatalytic formaldehyde oxidation. In comparison to nanoparticles, which can also be made into composite nanofibers, nanorods have increased delocalization of charge carriers, increasing the time before photogenerated charge carriers (electrons and holes) will recombine. The nanorods were thus investigated for increased photocatalytic reaction efficiency.

Shie *et al.* investigated the use of an ultraviolet light emitting diode (UVLED) as the light source to activate photodegradation of formaldehyde on TiO_2_ and Ag/TiO_2_ photocatalysts [58]. UVLEDs are available from several manufacturers with different wavelengths and intensities, and they have been investigated as alternative light sources for photolithography [59]. In the work of Shie *et al.*, each UVLED had a maximum luminous intensity at λ = 383 nm and 20 mW power consumption. They found that compared to both traditional 254 nm and 365 nm UV lamps, the UVLED had the highest energy effectiveness E_e_, defined as E_e_ = [decomposition mass of formaldehyde (mg)]/[input power (kWh)], while all of them had high decomposition efficiency η. The low cost and long lifetime of UVLEDs make them an attractive option for the development of low-power sensors for wide-area arrays.

### 2.3. Microscale Mechanical Sensors: MEMS Cantilevers for Mass Detection

**Figure 5.**
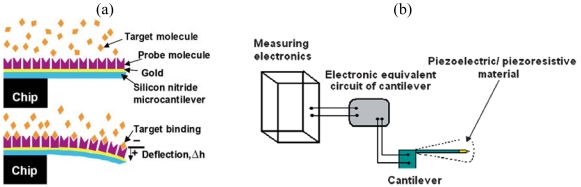
MEMS cantilever-based sensor. Target binding causes cantilever deflection, reprinted from [65] with permission from Macmillan Publishers Ltd, and piezoresistor detection, reprinted from [63] with permission from The Royal Society of Chemistry.

Microfabricated cantilevers have been used as sensors for detecting the presence of proteins, bacteria and viruses [60-62]. In the dynamic mode, the cantilevers can be used as sensors when molecules or cells adsorb onto the cantilever, increasing its mass and thus its resonant frequency. The cantilevers can also be used in static mode, when the adsorption of molecules onto the cantilever cause bending and result in surface stress (Figure 5a). The bending of the cantilever can either be detected using optical means to measure deflection of a beam, or using piezoresistors, which undergo a change in resistance [63] (Figure 5b). These cantilevers can be fabricated using the same process as for atomic force microscopy (AFM) cantilevers [64] but are usually functionalized with specific recognition (“probe”) molecules which will capture the “target” molecules from a mixture. The cantilevers are often coated with gold so that the probe molecules can be attached to the cantilever using thiol chemistry [65]. In the case of piezoresistive detection, the cantilevers can be integrated with microelectronics to produce a microscale sensor package.

Seo *et al.* used piezoresistive cantilevers as a formaldehyde sensor [66]. They found that cantilevers coated with 3-mercaptophenol had the largest downward deflection compared to 2-mercaptophenol, 4-mercaptophenol, and 1-mercapto-6-hexanol. The lowest detection limit was in the range of 0.027 ppm. While reaction of formaldehyde with the 3-mercaptophenol induced compressive stress in the cantilevers, the adsorption of benzene, toluene, and xylene produced tensile stress and upward bending of the cantilevers. However, Seo *et al.* found that the sensor curvature did not recover its original value after formaldehyde exposure and subsequent rinsing in air, indicating that the formaldehyde may have chemically reacted with the 3-mercaptophenol. Thus, regeneration of this sensor still requires further work.

## 3. Polymer-Based Sensors

Another promising approach for future development in formaldehyde gas sensors is the use of polymer-based sensing layers. They are good candidates because of the high sensitivity, short response times, reversibility and the capability to operate at ambient temperature [67]. Polymers, typically in the form of solid thin films, are used as the sensing medium in a variety of solid-state gas and fluid sensors since they can be easily fabricated, and their properties can be tailored for a given sensing application by careful selection of monomers and synthesis methods [68]. In many cases the VOC under investigation reacts directly with the polymer, altering its physical properties. The concentration of the VOC can then be related to this change, i.e. change in conductivity of an intrinsic conductive polymer or change in transmission spectrum. However many important volatile organic compounds are not or are only slightly reactive under mild conditions. Hence it is difficult to detect them by their chemical reactions with polymers. VOCs may have weak physical interactions with the polymers, such as adsorption or absorption, leading to a mass change or swelling of the polymer matrices [69] Absorption plays an important role in all the sensing techniques. Bartlett *et al.* presented a basic model for polymer gas sensors describing absorption kinetics [70]. Other ways to detect absorbance of the polymer matrix are based on piezoelectric sensors (quartz crystal microbalance and surface acoustic wave sensors), optical devices, which monitor changes in the absorption spectra, using UV or IR, or surface plasmon resonance, and amperometric sensors [69]. Another example of an optical sensor incorporates electrospun fluorescent polymer nanofiber membranes for fluorescence-quenching based detection of metal ions and 2,4-dinitrotoluene [71]. A way to increase the interaction with non reactive VOCs is to incorporate catalyst into the polymer matrix. The combination of organic/inorganic composites opens the door to more selective and sensitive sensors.

The adsorption of VOCs to the polymer will change its mass, which can be detected using piezoelectric crystal sensors in either quartz crystal monitor (QCM) or surface acoustic wave (SAW) devices. Poly-Lactic Acid-co-Glycolic Acid nanofibers have been deposited on thickness shear mode piezoelectric sensors [72,73]. Feng *et al.* have developed a formaldehyde sensor using a molecularly imprinted polymer as the recognition element and QCM as the sensor [74]. Molecularly imprinted polymers have been used as antibody mimics in recognition systems [75], enzyme mimics for catalytic applications [76], and recognition elements in biomimetic sensors [77]. Compared to biomolecules such as antibodies or nucleic acids, which can recognize targets with high affinity but have poor chemical stability, molecularly imprinted polymers have been used due to their robustness in different various environments (organic solvents, pH, temperature). Molecularly imprinted polyurethanes have been used with QCM to detect chloroform, ethanol, and other solvents [78]. Feng *et al.* used a non-covalent method to imprint formaldehyde template molecules in methacrylic acid, which was polymerized directly on the quartz crystal monitor. Compared to non-imprinted polymer, the imprinted polymer demonstrated selectivity for formaldehyde, whereas other contaminants such as benzaldehyde and acetone were not clearly distinguished between the two sensors.

### 3.1. Conducting Polymer Based Sensor

**Figure 6.**
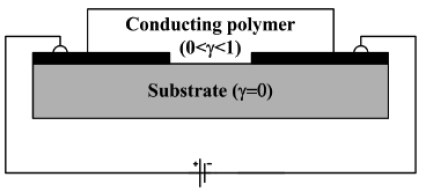
Chemiresistor configuration: The electrical resistance change resulting from a change in the gas concentration is measured between two electrodes [69], where *γ* is the normalized gas concentration.

The most widely used sensor configuration is the chemiresistor (Figure 6). A chemiresistor consists of one or several pairs of electrodes and the electrical resistance change is measured at the output. A simple ohmmeter suffices to collect the data. Several models have been developed to correlate the normalized gas concentration *γ* in the ambient to the concentration inside the conducting film [79]. Other often used configurations include organic thin film transistors OTFTs [80,81], or diode type sensors [82], which require more fabrication steps.

Recently a new class of polymers known as intrinsically conducting polymers or electroactive conjugated organic polymers has been increasingly applied in sensing materials based on the unique electrical and optical properties [83]. The charge transfer mechanisms differ fundamentally from inorganic crystalline semiconductors such as silicon, in that they are molecular in nature. A polymer becomes intrinsically conductive if the molecular orbitals overlap, allowing a delocalized wave function. The free movement of the charge carriers throughout the lattice is maximized when the orbitals are only partially filled. To describe the electronic phenomena the concept of solitons, polarons and bipolarons have been introduced [84]. The most widely used intrinsically conductive polymers for sensor applications are polypyrrole (PPy), polyaniline (PANi), polythiophene (PTh) and their derivatives. Although the pure polymers may have relatively low conductivities (<10^–5^ S/cm^–1^[69]) they can be reversibly doped through oxidation or protonation. Conductivities in the range of semiconductors or conductors (∼10^0^–10^5^ S/cm^–1^) can be achieved in the doped polymers. The detection limits for conducting polymer sensors can be <1 ppm for acid-base active analytes, and on the order of several ppm for inert organic analytes, with response times on the order of seconds for ultra thin film sensors [69].

**Figure 7.**
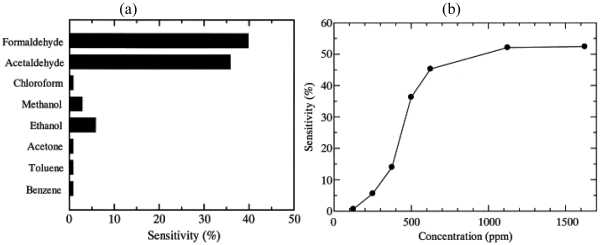
(a) Sensitivity of PPy/EBSA films at room temperature upon exposure to different analytes (500 ppm) and (b) the change of sensitivity as function of analyte concentration, reprinted from [85] with permission from Elsevier.

Hosono *et al.* synthesized a highly conducting PPy thin film by plasma polymerization [85]. They then doped the polymer using 4-ethylbenzenesulfonic acid (EBSA) as reported earlier [86] resulting in three orders of magnitude larger conductivity than iodine-doped PPy films. They define the sensitivity as (R_air_-R_gas_)/R_air_ × 100(%), in which R_air_ and R_gas_ were the electrical resistances when exposed to air and gas respectively. In Figure 7(a) the sensitivity to different gases is reported. The group suggests that the conductivity change is mainly caused by the increased charge carrier concentration in the polymer backbone. They reported as well sensitivity changes as a function of analyte concentration Figure 7(b) and humidity.

The sensitivity and time response of the sensing film is in general proportional to the surface area of the film per unit mass. Changing the morphology at the nanoscale offers therefore the possibility to increase the performance of the sensor. Nanofiber-based sensing films have one to two orders of magnitude larger sensing area per unit mass than solid films [87] and therefore offer faster response times and increased sensitivity. Polymer nanofibers have been prepared using interface polymerization [88], surfactant methods [89], as well as electrospinning [87]. Of these nanofiber production methods, electrospinning offers easily controllable fibre diameter and high uniformity using a relatively simple fabrication system. An electric field is applied between the polymer solution reservoir and a collection ground plate. The stretching forces induced on the jet by the electric field and the rapid evaporation of the solvent causes the formation of solid nanofibers during the passage from the tip to the collection screen. Polymer nanofibers have been interfaced with microfabricated structures [90] and the characteristics of single fibers are investigated. More than one hundred different polymers, polymer blends, and polymer-nanoparticle composites have already been electrospun into nanofibres, summarized in a recent summary of gas sensors based on electrospun nanofibers [87].

### 3.2. Polymer Nanocomposites for Sensors

Alternatively, conductive polymers can be used as the organic fraction of organic/inorganic hybrids. Zheng *et al.* [91] used a PANi-TiO_2_ nanocomposite as the active layer on a QCM sensor. They dispensed an aqueous solution containing PANi and TiO_2_ nanoparticles directly onto the electrodes and allowed it to dry. They reported response times of less than 300 s and they showed the response to formaldehyde and several other VOCs (Δf ≈ 100 Hz for 150 ppm of formaldehyde). They report as well that the addition of TiO_2_ improved the thermal stability of the polyaniline sensor. Ma *et al.* [92] showed that the size and shape of the oxide particles, degree of dispersion, kind of interaction between the organic and inorganic phase, as well the material of the electrode and the concentration of the analyte itself are all factors which affect the response of the sensor.

Another class of organic/inorganic hybrids is synthesized by an intercalation reaction. Matsubara *et al.* interleaved PPy chains with molybdenum oxide (MoO_3_) layers through ion exchange [93]. The structure of pure MoO_3_ consists of layered, covalently bonded double-octahedral oxide sheets held together by Van der Waals forces. A variety of large organic compounds can be intercalated between the layers [94]. The conductive nature of the PPy chains is responsible for the drastic resistivity decrease from 2 × 10^10^ Ω-cm of pure MoO_3_ to 9.6 Ω-cm of the PPy doped MoO_3_ layers. The charge carrier transport properties are governed by the host layer (MoO_3_) but the magnitude of resistivity is dependent on the corresponding polymer. A detailed study on the sensing mechanism is still in progress. Matsubara *et al.* suggest that the analyte has two effects on the resistivity of the hybrid material. The first is the partial electron transfer between the analyte and the polymer causing a change in charge carrier density in the polymer backbone. The second is a physical effect due a change in the interlayer distance affecting the charge transfer between the two different materials. The response to a formaldehyde exposure of 500 ppm was up to 6% with response times from 90–120 s. The group further studied other organic/MoO_3_ hybrid systems and improved their response to formaldehyde [95]. They found that the PPy/MoO_3_ and PANi/MoO_3_ exhibited 4-8% change in conductivity to an exposure of 50 ppm of formaldehyde. They concluded that the sensitivity is limited by large insoluble PANI moieties adsorbed onto the hybrid surface. PANI shows an opposite response compared to the hybrid. They modified the intercalation process accordingly by eliminating the insoluble polymer residues [96,97]. With their new sensing layers they achieved a 4% change in conductivity when exposed to 200 ppb.

## 4. Conclusions

Continuous industrial monitoring of volatile organic compounds will have a large impact in improving public health worldwide. As a VOC, formaldehyde will continuously outgas from manufactured wood products such as furniture or other household materials, and these fumes can be trapped inside buildings. Sustained exposure to formaldehyde levels as low as 80 ppb can lead to adverse health effects. Miniaturization of formaldehyde sensors promises to address some of the challenges to wide-scale deployment of sensor arrays for real-time residential and industrial monitoring. Microfabricated devices such as microhotplates for metal oxide sensors offer reduced power consumption and increased portability and ease of use compared to laboratory techniques. The reported detection limits for several microfabricated formaldehyde gas sensors are summarized in Table 1.

Polymer and polymer composite sensor materials have the potential to offer further advantages, in room-temperature operation and increased sensitivity or selectivity. Novel approaches to monitoring the presence of volatile organic compounds such as formaldehyde will combine new composite materials (nanoparticle catalysts and conductive polymer nanofibers), which provide sensing capability for the presence of environmental contaminants, and MEMS and microsystem platform technologies to provide electrical interconnect to the new nanomaterials. The goal is to design robust manufacturing methods to ensure that eventual large-scale production and use of the integrated sensor is possible.

## Figures and Tables

**Table 1.** Microfabricated formaldehyde gas sensors.

**Technology**	**Detection limit / Sensitivity**	
**Metal Oxides**		

NiO film, microhotplate	Detection limit 0.8 ppm at 300 °C, in air	[43]
SnO_2_-NiO film, microhotplate	0.06 ppm, 180 mW at 300 °C, in air	[28]
NiO film, ceramic plate substrate	0.825 mV/ppm, in dry air	[27]
Li-doped NiO film, ceramic plate structure	0.488 mV/ppm, in dry air	[27]
Porous SnO_2_, ceramic plate structure	(R_air_–R_gas_)/R_air_ = 10 at 300 °C for 100 ppb	[34]
NiO film, microhotplate	Detection limit 1.2 ppm at 280 °C	[42]
NiO/Al_2_O_3_ cosputtered, microhotplate	-0.137 Ω/ppm at 280 °C, in air	[44]
	- 0.335 Ω/ppm at 280 °C, increased active area	
	Detection limit 40 ppb	
ZnO/ZnSnO_3_, ceramic tube structure	R_gas_/R_air_ = 34.5 to 50 ppm at T unknown, 5 V on heating wire, in air	[32]
CdO-In_2_O_3_, ceramic tube structure	R_gas_/R_air_ = 80 to 10 ppm at 133 °C, air	[26]
SnO_2_-In_2_O_3_-CdO, ceramic tube structure	R_gas_/R_air_ = 559 to 300 ppm at 133°C, in air	[37]
LaFe_1-x_Zn_x_O_3_, ceramic tube structure	LaFe_0.77_Zn_0.23_O_3_ highest sensitivity, R_gas_/R_air_ = 44.2 to 100 ppm at 240 °C, in air	[35]
La_x_Pb_1-x_FeO_3_, ceramic tube structure	La_0.68_Pb_0.32_FeO_3_ highest sensitivity, R_gas_/R_air_ = 9 to 500 ppm at 180 °C, in air	[36]
SnO_2_ doped with MWCNTs 5wt%	Lowest detection of 0.03 ppm at 70 °C, in air	[8]
Photocatalytic, ZnO nanorods	Detection limit 1.78 ppm at 20 °C, in air, 30 % rel. humidity	[57]
**Mechanical detection principle**		
piezoresistive microcantilever	Detection limit 0.027 ppm	[66]
QCM, molecularly imprinted polymer	20.5 μM, N_2_	[74]
**Enzyme based**		
FALDH amperometric, Teflon membrane	1.9 μA/ppm, detection limit 130 ppb, in air	[13]
FALDH amperometric, ceramic membrane	0.5 μA/ppm, detection limit 76 ppb, in air	[17]
**Polymer – based sensors**		
PPy/EBSA	40% resistance change upon 500 ppm at 20 °C, in air	[85]
PANi-TiO_2_, QCM	Δf = 100 Hz for 150 ppm, in air	[91]
PPy/MoO_3_ and PANi/MoO_3_	4–8% conductivity change upon 50 ppm, in N_2_	[95]
PPy/MoO_3_ and PANi/MoO_3_	2–5% conductivity change upon 100 ppb–500 ppb, in N_2_	[97]
